# Contraceptive Access at Federally Qualified Health Centers During the South Carolina Choose Well Initiative: A Qualitative Analysis of Staff Perceptions and Experiences

**DOI:** 10.1089/whr.2021.0060

**Published:** 2021-12-15

**Authors:** Liane M. Ventura, Kate E. Beatty, Amal J. Khoury, Michael G. Smith, Oluwatosin Ariyo, Deborah L. Slawson, Amy J. Weber

**Affiliations:** ^1^ Department of Health Services Management and Policy, Center for Applied Research and Evaluation in Women's Health, East Tennessee State University, Johnson City, Tennessee, USA.; ^2^ Department of Community and Behavioral Health, College of Public Health, East Tennessee State University, Johnson City, Tennessee, USA.

**Keywords:** Choose Well, contraception, family planning services, federally qualified health centers, Health Services Accessibility, safety-net providers

## Abstract

***Introduction:*** Federally qualified health centers (FQHCs) provide essential contraceptive services to low-income individuals; yet, access to all method options, notably intrauterine devices (IUDs) and implants, may be limited at non-Title X FQHCs. The South Carolina (SC) Choose Well initiative is a statewide contraceptive access initiative that was launched in 2017 and extends into 2022. Choose Well established a collaborative network between training and clinical partners and is aimed at facilitating implementation of contraceptive care best practices through capacity-building and training of clinical and administrative staff in partner organizations. The initiative provided funding for workforce expansion and contraceptive methods. We examined perceptions of staff from Choose Well-participating FQHCs regarding contraceptive access during the first 2 years of the initiative, including factors that facilitated or posed access challenges as well as sustaining factors. This study informs the process evaluation of Choose Well while providing data critical for uncovering and scaling up contraceptive access initiatives.

***Materials and Methods:*** Interviews were conducted with FQHC staff (*n* = 34) in 2018 and 2019 to assess Choose Well implementation and were recorded, transcribed, and double-coded *via* at least 80% interrater reliability or consensus coding. Data were analyzed according to clinical and administrative factors influencing contraceptive access.

***Results:*** Increased capacity for contraceptive counseling and provision through training and external funding for IUDs and implants were the most noted clinical factors facilitating access. Streamlining workflow processes was also a facilitator. Buy-in and engagement among staff and leadership emerged as a facilitator at some clinics and as a barrier at others. Policy/structural factors related to costs of devices and insurance coverage were identified as threats to sustainability.

***Conclusions:*** The Choose Well initiative contributed to the perception of an increase in contraceptive access at participating FQHCs in SC. Statewide contraceptive access initiatives have the potential to support FQHCs in meeting their clients' contraceptive needs. Organizational buy-in, sustainability of funding, and training are key to realizing the full potential of these initiatives.

## Introduction

More than 7 million women at risk of unintended pregnancy obtain contraceptive services from publicly funded safety-net clinics nationwide.^1^ Key to supporting the reproductive autonomy of these women is contraceptive counseling that honors their needs and preferences and access to the full range of contraceptive methods.^2^

However, access to the full range of methods, including intrauterine devices (IUDs) and implants, is restricted by a multitude of barriers such as high upfront cost,^3^ required return office visit for placement of the device,^4^ and limitations in the number of providers who are trained and/or willing to provide IUDs and implants.^5–7^

Access to contraceptive methods is complex; the dimensions of health care access provide a framework to assess the interrelated components of availability (supply and demand), accessibility (location), accommodation (organization of resources), acceptability (appropriateness), and affordability (cost).^8,9^

Due, in part, to funding mechanisms, not all publicly funded safety-net clinics provide equal access to the full range of contraceptive methods.^10^ Federally qualified health centers (FQHCs) are an essential component of the nation's health care safety-net, but not all FQHCs provide on-site contraceptive care. While federal law requires FQHCs to provide preventive and primary care, including contraceptive care, FQHCs can offer contraceptive services directly or through agreements with other providers. Also, an FQHC system may offer contraceptive care at some, but not all, of its service delivery sites.

In addition, FQHCs vary in the range of contraceptive services offered, with some offering limited services (*e.g.*, screening and treatment for sexually transmitted infections and one or two contraceptive methods) and others offering more comprehensive care.^10^ Center characteristics, the policy environment, and Title X funding influence the range of services offered.

Title X is the sole federal funding mechanism specifically allocated for the provision of contraceptive services,^11^ and providers, including health departments, family planning clinics, hospitals, and some FQHCs,^12^ rely on Title X funding to provide contraceptive care.^13^ In South Carolina (SC), FQHCs do not receive a portion of the ∼$6 million statewide Title X funding,^14^ as Title X funding is distributed through the state health department.

FQHCs that do not receive Title X funding are less likely to have on-site availability of contraceptive methods, such as stocking IUDs and implant devices,^3,15^ and are less likely to offer contraceptive services that require additional training, such as IUD and implant placement and removal procedures, than Title X-funded family planning clinics.^16–18^ Without comprehensive clinical practice guidelines and Title X funding, there is considerable variability in contraceptive service provision among FQHC clinics.^10^

Despite funding limitations, the number of contraceptive care patients served by publicly funded clinics that do not receive Title X funding nationwide increased by 29% between 2010 and 2016.^1^ As FQHC's role in delivering contraceptive care within the publicly funded safety-net grows, it is increasingly important to support these clinics and to examine perceptions of staff regarding facilitators and barriers to contraceptive access at their clinics.

Nationwide, federal, and state grant funding has facilitated contraceptive provision at FQHC clinics.^19^ In addition, contraceptive access initiatives promise to expand access to contraceptive care at partner organizations. In SC, Choose Well is a statewide initiative, which is funded by a private philanthropic foundation, aimed at expanding access to contraception and reducing unintended pregnancy. The 6-year initiative launched in 2017 and extends into 2022. In 2017, a total of 20 FQHC systems in SC offered family planning services at 123 clinic sites across the state, and of these, 8 systems and 33 clinics participated in Choose Well. By 2018, additional FQHC systems and clinics had joined Choose Well bringing the number of participating systems to 16 and the number of participating clinics to 57.

In 2017 and 2018, participating FQHCs received funding and support through Choose Well to bolster contraceptive counseling and service provision. Participating FQHCs received capacity building and provider and administrative trainings (in the areas of contraceptive counseling, IUD and implant placement and removal, billing and coding for contraceptive services, revenue cycle, and inventory management); clinic infrastructure and workforce enhancements, including funding for contraceptive methods and staff positions, and technical assistance opportunities.^20,21^

As a part of the external evaluation of Choose Well, we assessed progress in implementation of the initiative at participating FQHCs during the initial 2 years with a focus on the availability of IUDs and implants. The *availability* dimension of access refers to components of supply and demand^22^ and explores a specific area of fit between service providers and patients.^8^ This construct includes providers who are trained and willing to place and remove IUDs and implants, on-site stocking of devices, administrative support for billing and coding for contraceptive services, and enhanced workflow processes.^9^

The study aim was to assess changes in contraceptive access associated with the Choose Well initiative and to identify facilitators and challenges to the provision of IUDs and implants as well as perceptions of sustainability of funding from the perspective of clinical and administrative staff at Choose Well participating FQHCs. This information is important for the process evaluation of Choose Well, and more broadly, to facilitate the translation of evidence-based interventions into practice. Specifically, identifying facilitators and barriers within the *availability* dimension of access, at the organizational and policy/structural levels, will inform prioritizing areas for improvement within FQHC systems/clinics as well as advocacy efforts for policy change, thereby helping to optimize the impact of statewide initiatives on clinical care among FQHCs. This, in turn, will improve experiences with care and reproductive outcomes for FQHC patients.

## Materials and Methods

### Data collection

Semistructured key informant interviews were conducted from July 2018 to September 2019 with Choose Well participating FQHC staff at the clinic- and system-levels. FQHCs are large nonprofit organizations composed of multiple delivery sites,^23^ with administrative staff working at the system/organizational level and clinic staff working to directly support or provide services at the individual delivery sites. The first round of interviews was conducted in 2018 and assessed staff perceptions of Year 1 (2017) implementation of Choose Well. In 2017, 33 FQHC clinics across 8 FQHC systems participated in Choose Well. In 2018, we invited 38 clinic-level staff (representing 32 of the 33 participating clinics; 1–3 individuals per clinic) and 11 system-level staff (representing all 8 participating systems; 1–3 individuals per system) to interview.

Of those invited, 13 clinic-level staff (34.2%) (representing 13 clinics) and 6 system-level staff (54.5%) (representing 4 systems) completed the first round of interviews.

The second round of interviews was conducted in 2019 and assessed staff perceptions of Year 2 (2018) implementation of Choose Well. In 2018, 57 clinics across 16 FQHC systems participated in Choose Well. In 2019, we invited 51 clinic-level staff (representing 39 of the 57 participating clinics; 1–3 individuals per clinic) and 21 system-level staff (representing 12 of the 16 participating systems; 1–3 individuals per system) to interview.

Of those invited, 18 clinic-level staff (35.3%) (representing 14 clinics) and 8 system-level staff (38.1%) (representing 7 systems) completed the second round of interviews. Across both rounds of interviews, 31 clinic-level interviews and 14 system-level interviews were completed. Of the 31 clinic-level interviews, 8 staff were interviewed in both rounds and counted once, generating a final sample of 23 clinic staff. Of the 14 system-level interviews, 3 staff were interviewed in both rounds and counted once, generating a final sample of 11 system staff, and a total of 34 staff at both levels over the study period (see Appendix Table A1 for details).

FQHC staff invited to interview were identified from lists provided by the Choose Well initiative and included staff engaged in or knowledgeable about Choose Well implementation at the system- or clinic-level.

At the clinic-level, we invited the clinic administrator and/or lead family planning provider (*e.g.*, contraceptive counselor, family nurse practitioner, other nursing staff). At the system-level, we invited Executive Directors, Directors of Operations/Finance, and Chief Medical Officer/Chief Nursing Officers.

Study staff iteratively developed semistructured discussion guides, which included open- and closed-ended questions. Interview questions assessed staff perceptions of the initiative's implementation in their clinic or system. All respondents were asked the same interview questions, although wording was adapted for clinic- and system-level staff to assess implementation at the clinic- or system-level, respectively.

Respondents were recruited using a multimodal approach, which included five stages of initial and follow-up e-mails and phone calls. All interviews were conducted over the phone by study staff and audio recorded with permission of the participants. Audio recordings were transcribed by a third-party vendor. Participants received a $50 electronic gift card after completing the interview. This study was approved by the medical Institutional Review Board at East Tennessee State University.

### Coding and analysis

Interview transcripts containing responses about contraceptive provision, training opportunities, and perceptions of sustainability of funding were independently coded using an iterative coding process between two coders. A codebook based on the interview guide was developed after initial review of transcripts^24^ and then refined after each round of coding through consensus coding. Emergent themes were clearly defined in the codebook.^25^ A minimum of 80% interrater reliability was applied for all coding.^26^

This dataset contains an aggregate of 2 consecutive years of interview data. In counting responses within emergent themes, responses from each individual were counted as one total, regardless of whether an individual answered in 1 year or in both years. Themes were analyzed without regard to clinic- or system-level responses, but quotes from each level were highlighted, respectively, in data tables. The coding was conducted with NVivo 12.0 software (released in March 2018).

**FIG. 1.**
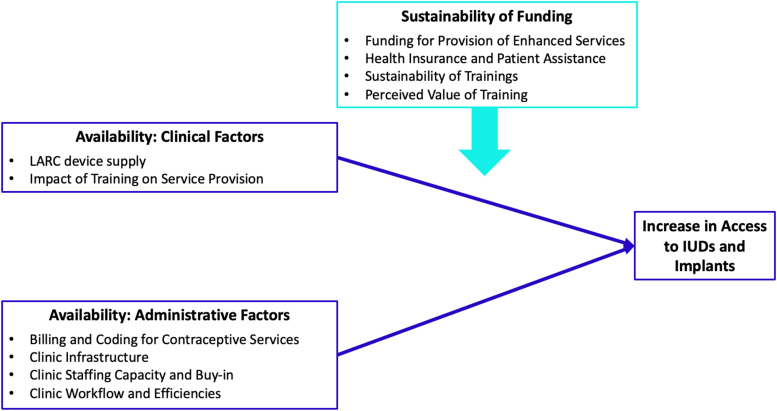
The theoretical model utilized to guide qualitative data analysis was based on the *availability* component of the health care access framework. Within the context of FQHC clinics and the Choose Well Evaluation, three key constructs (Availability: Clinical Factors; Availability: Administrative Factors; Sustainability of Funding) were hypothesized to influence contraceptive access. Through an inductive exploration of the interview data, emergent factors/themes impacting access were identified and then mapped to each of these three constructs. In addition, through an inductive analysis of the data, the relationships among the three constructs relative to contraceptive access were identified. Specifically, two of the three constructs (Clinical Factors and Administrative Factors) were found to directly contribute to an increase in access to implants and IUDs, whereas one construct (Sustainability of Funding) appeared to influence the identified clinical and administrative factors that impacted access. The conceptual constructs, emergent themes within constructs, and relationships relative to contraceptive access are illustrated in Figure 1. FQHC, federally qualified health center; IUDs, intrauterine devices.

The *availability* dimension of health care access,^22^ operationalized in terms of three main constructs (Availability: Clinical Factors; Availability: Administrative Factors; Sustainability of Funding) offered a framework for analysis (Fig. 1). Emergent themes influencing availability of IUDs and implants, as identified during the analysis, were mapped to these three constructs.^9^

The themes within clinical or administrative factors were subsequently aggregated into broader themes through a team-based data consolidation approach.^24^ Representative quotations for each theme were selected to highlight perspectives of clinic- and system-level respondents.

## Results

This study examined perceptions of FQHC staff regarding organizational factors, including clinical and administrative factors, which influenced access to IUDs and implants at FQHC clinics in SC during the initial 2 years of the Choose Well initiative. We also examined perceptions of sustainability, including foreseen facilitators and challenges.

### Respondents

Respondents at the clinic-level held a variety of roles, including Certified Medical Assistant, Clinical Support Supervisor, Case Manager, Family Nurse Practitioner, Practice Manager, and Reproductive Health Manager. Respondents at the system-level included Chief Executive Officer, Chief Quality Officer, Clinic Operations Director, Director of Patient Services, and Project Manager.

### Clinical factors

Two themes emerged related to clinical factors influencing contraceptive access at participating FQHCs during the Choose Well initiative: IUD and implant device supply; and impact of training on service provision (Table 1).

**Table 1.** Availability—Clinical Factors

Device supply		
Facilitators	Total (*N* = 34)	Representative quotations
External funding for IUDs and implants	22	“Having that funding to be able to have the devices in-house is the biggest thing for us.” [Clinic level]
		“[External funding] offers to pay for certain long-term methods that our patients would never have been able to afford otherwise.” [System level]
Ability to provide same-day placement procedures	17	“Providing same-day access to IUDs and [implants] is a real positive thing for our patients.” [Clinic level]
		“We tripled our numbers in 2018 from 2016 and 2017. [External funding has] given us the opportunity to have on hand what we need when we need it. Seize the moment.” [System level]
Stockpile of devices on-site	10	“[External funding] helped us stockpile [IUDs and implants]” [Clinic level]
		“We stock the devices and they are onsite, make them readily available.” [System level]

Training for IUD and implant provision and contraceptive counseling		
Facilitators	Total (*N* = 34)	Representative quotations
Increased capacity for and enhanced contraceptive counseling	24	“…We're able to offer counseling services as well, which is an entity that was not existent at all. I think that's a great part of family planning, especially for teenagers who for some reason feel that they should get pregnant, it gives them the opportunity to talk to someone about it.” [Clinic level]
		“[Trainings have] made our staff more comfortable in having those conversations, our providers as well. Some of them tend to be a little bit hesitant to talk about birth control.” [System level]
Increased overall clinical capacity to provide contraceptive care	19	“Overall performance, we see more patients of course, and we're offering more of reproductive health and also our numbers have gone up significantly, on their numbers especially for long-term birth controls. The clinic has definitely seen that growth in that area.” [Clinic level]
		“… We know that it has brought in more revenue because [the implant] is a very expensive contraceptive device. Of course, given that we are a federally qualified health center, we take that revenue and use that revenue to provide more services.” [System level]
Increased provider capacity for IUD and implant provision	19	“Some of the strengths of the training were the ability to truly see the placement of [IUDs and implants] The whole training made providers more confident in [IUD and implant] insertion.” [Clinic level]
		“We have more providers now that can offer [IUDs and implants], can do insertions and removals of [IUDs and implants]”. [System level]
Patient education and outreach as an element of counseling	17	“Sitting there, being able to explain to a patient and then you actually show them what the birth control looks like and everything. I think that has definitely helped patients in making a more informed decision.” [Clinic level]
		“All the topics that have been covered around how to present the devices, how to present education around contraceptive access has helped our staff be a little more proactive in making those conversations happen and also be able to answer questions that patients have when they get asked.” [System level]
Expanded perspective on reproductive health	11	“The trainings in which I participated were really great, especially the ones that dealt with your thoughts surrounding different reproductive health issues. There were some very good exercises that forced you into looking at points of view that you might not necessarily share concerning reproductive health, sexuality, and different things of that nature that I thought were really eye-opening…” [Clinic level]
		“…some people aren't receptive or open to talking about family planning methods other than traditional methods. That was eye opening and it did create dialog, so that was great.” [System level]
Improved quality of contraceptive care services through training	9	“[Training] enhances the quality of care that the patients have been receiving…” [Clinic level]
		“[Training has] definitely improved our family planning education for patients.” [System level]

Barriers		
Provider factors	4	“Not every provider is comfortable with doing an IUD. They might be okay with a [an implant], but they're not all comfortable with doing an IUD…” [Clinic level]
		“It's not their fault, but I have providers who have strong beliefs and that's always going to be a barrier.” [System level]

IUDs, intrauterine devices.

Discussing device supply, the majority of respondents indicated that an increase in access to IUDs and implants was facilitated by external funding, ability to provide same-day IUD and implant placement procedures, and on-site stockpiling of devices. For example, “Having that funding to be able to have the devices in-house is the biggest thing for us.”

Training opportunities provided through the initiative were noted to have enhanced and increased capacity for contraceptive counseling, increased overall clinical capacity to provide contraceptive care, and specifically increased provider capacity for IUD and implant provision. One respondent noted, “We have more providers now that can offer [IUDS and implants], can do insertions and removals of [IUDs and implants].”

In addition, half of the respondents highlighted the benefits of training for patient education and community outreach as a component of contraceptive counseling. Respondents also emphasized the benefits of an expanded perspective on reproductive health, such as “gaining viewpoints,” “becoming more open,” and “creating dialog” about contraceptive methods, sexuality, unintended pregnancy, and contraceptive counseling. Improved quality of contraceptive care services was also noted, “[Training] enhances the quality of care that the patients have been receiving… ”

However, provider factors (beliefs and comfort level) emerged as a barrier among a few respondents, for instance, the perception that some providers were “not comfortable with doing an IUD” or “have strong beliefs that's always going to be a barrier.”

### Administrative factors

Four themes emerged related to administrative factors influencing contraceptive access, including the provision of IUDs and implants, during the Choose Well initiative: billing and coding for contraceptive services; clinic infrastructure; clinic staffing capacity and buy-in; and clinic workflow and efficiencies. Facilitators were identified across all themes, and barriers were identified in three domains (Table 2).

**Table 2.** Availability—Administrative Factors

Billing and coding for contraceptive services		
Facilitators	Total (*N* = 34)	Representative quotations
Maximizing reimbursement and increasing revenue potential	5	“We're also looking at how our providers are billing and coding for family planning services, making sure that they maximize reimbursement so that we can continue the initiative moving forward without Choose Well funding.” [Clinic level]
		“…From a revenue generation perspective, the services pay for themselves. So we can use their revenue to sustain the program.” [System level]
Improvements in billing and coding practice	4	“I definitely think we're learning more about billing and trying to beef up our billing” [Clinic level]
		“As far as billing, they're more mindful of how things are being coded.” [System level]

Barriers		
Lack of knowledge about appropriate billing and coding	5	“We've experienced some systems challenges related to coding, having our providers be able to code correctly for family planning services.” [Clinic level]
		“I think we have ongoing billing challenges. I think there's ongoing documentation and billing challenges.” [System level]

Clinic infrastructure		
Facilitators	Total (*N* = 34)	Representative quotations
Robust improvement of Electronic Medical Record (EMR)/Electronic Health Record (EHR) systems	6	“We now have templates in place for when we do IUDs and [implants]”. [Clinic level]
		“We're trying to do additional training and general upgrades in the EMR.” [System level]
Improved quality of clinic infrastructure	2	“Definitely our infrastructure had to change here because the insertion of a [an IUD or implant] is not the same as a 15-minute visit.” [Clinic level]
		“[External funding] has helped with the family planning infrastructure.” [System level]

Clinic staffing capacity and buy-in		
Facilitators	Total (*N* = 34)	Representative quotations
Buy-in and engagement among staff and leadership	13	“As new providers are hired on that as part of the interview process, what their feeling is regarding performing these types of procedures within the setting here at [Health Care Center] so that we can continue to sustain what we've accomplished.” [Clinic level]
		“Our CEO is very supportive. I would think she wants to see [the program] continue and succeed.” [System level]
Expanded clinic workforce	8	“The [IUD and implant] supply and I would say the additional funding for the provider.” [Clinic level]
		“I think that the biggest thing that [external funding] has done for us, is allow us to have a dedicated team.” [System level]

Barriers		
Lack of buy-in to organizational change	8	“There's some barriers still where [some providers] think that [implants] are okay, but for an adolescent, an IUD, they're not on board with. We're still working on changing some mindsets.” [Clinic level]
		“It took a little while to get going with staff buy-in from the support staff.” [System level]
Workforce turnover	5	“Then finding someone with experience to be patient educator was difficult.” [Clinic level]
		“My team had talked about [turnover] with the CEO, but we have had some challenges at [Health Care Center] in that we have lost several of our Champion providers. We have new providers who are coming in this fall and we'll train them and assess their level of comfort and their desire.” [System level]

Clinic workflow and efficiencies		
Facilitators	Total (*N* = 34)	Representative quotations
Enhanced clinic workflow processes	12	“We realized that the plan that we had, what we were currently doing was not successful. We're wanting to change that. Everybody is very optimistic about moving forward and seeing how this new workflow works for us.” [Clinic level]
		“With some of the training on when the referrals come over, we know how to assess our schedule appropriately, to try to make sure we get this person in fairly quickly.” [Clinic level]
Institutionalizing changes into the current system	4	“The teenage pregnancy question is part of our workflow so it prompts various other things for me as the [primary health care provider] as well so I think that will continue to be a huge part of what we do.” [Clinic level]
		“[Reproductive services are] part of our daily delivery now.” [System level]
Tracking and utilizing clinic data	4	“Right now, we're really looking forward to receiving data, so that the data can help us tell our story when we're going to other funders to try to help sustain the program.” [Clinic level]
		“I now have trended how many we are putting in at every site. I'm able to know on a quarterly basis now, I've ordered all of our supplies, all of our methods… Outside of this grant I have a reproductive healthcare budget as well here at [Health Care Center].” [System level]

Barriers		
Challenges to updating clinic infrastructure	4	“I think the challenge was getting the sexual health assessment in place from an IT perspective or a system enhancement perspective, getting it in place.” [Clinic level]
		“It took us nine months to almost a year to figure out every single thing that we needed at every single site.” [System level]
Tracking supplies and methods	1	“That was my biggest challenge was tracking everything from equipment to supplies to methods.” [System level]

Related to billing and coding, respondents noted the importance of maximizing reimbursement and increasing revenue potential for contraceptive services. As one respondent noted, “From a revenue generation perspective, the services pay for themselves.…” While some respondents indicated that improvements in billing and coding practice at their clinic(s) had facilitated IUD and implant provision, others indicated that lack of knowledge about billing and coding was a challenge.

Regarding clinic infrastructure, robust improvement to electronic medical record or electronic health record systems was noted as a facilitator by some respondents, such as adding templates and conducting upgrades.

In discussing staff capacity and buy-in, several respondents noted that buy-in and engagement among both staff and leadership and expanded clinic workforce had facilitated access to IUDs and implants at their clinics. For example, “Our CEO is very supportive. I would think she wants to see [the program] continue and succeed.” Yet, several other respondents noted lack of buy-in to organizational change and workforce turnover as administrative barriers to increased access. Regarding a lack of buy-in: “There's some barriers still where [some providers] think that [implants] are okay, but for an adolescent, an IUD, they're not on board with. We're still working on changing some mindsets.”

Enhanced clinic workflow processes, institutionalizing changes into the current system, and tracking and utilizing clinic data were indicated as facilitators of service provision. One respondent highlighted “[reproductive health services are] part of our daily delivery now.” A few respondents, however, noted challenges to updating clinic infrastructure along with difficulties in tracking supplies and methods.

### Sustainability of funding

Sustainability of funding was examined given its importance in maintaining changes associated with Choose Well implementation. This construct included four domains: funding for provision of enhanced services; health insurance and patient assistance; sustainability of trainings; and perceived value of training (Table 3).

**Table 3.** Sustainability of Funding

Funding for provision of enhanced services		
Facilitators	Total (*N* = 34)	Representative quotations
340B drug pricing program	5	“We are a 340B FQHC. This helps us provide contraceptives to clients whose insurance doesn't cover it.” [Clinic level]
		“We're a 340B entity, so we can get [IUDs and implants] at 340B prices. That really helps a lot.” [System level]
Other external funding to provide IUDs and implants	5	“[Leadership] has some ideas as far as obtaining funding so that we would be able to order devices and have them in stock ahead of time.” [Clinic level]
		“Part of our sustainability is maybe try to find other funding.” [System level]

Barriers		
High cost of devices	12	“… Unfortunately, 60 percent of our patients are uninsured, and I am not certain how we're going to fund IUDs and [implants] for patients who are uninsured [after external funding ends].” [Clinic level]
		“Once the money goes away to buy devices, then we're not going to be able to offer free devices anymore.” [System level]

Health insurance and patient assistance		
Facilitators	Total (*N* = 34)	Representative quotations
Increased number of patients enrolling in Medicaid	6	“Getting as many of our patients signed up on family planning Medicaid as possible is the ticket.” [Clinic level]
		“You're going to get the reimbursement from the insurance companies and from the Medicaid. We're trying to get everybody that will qualify on Medicaid, on Medicaid, at least for family planning if they don't qualify for the full Medicaid.” [System level]
Patient assistance programs	6	“As far as billing-wise, since we deal with a large uninsured population, we have programs to help them pay for their services at a low cost to them.” [Clinic level]
		“[One of the pharmaceutical companies] do offer a patient assistance program. That would be a good thing for our patients who can't really afford it, a patient assistance program.” [Clinic level]
Sliding scale fee at clinics	3	“We do offer them a sliding scale fee. It's a certain amount that patients have to pay when they're uninsured, like as low as $20.” [Clinic level]
		“We already had the sliding fee.” [System level]

Barriers		
High rate of uninsured patients	6	“We have the highest uninsured population of all health centers in the state of South Carolina. It's where we are in our growth, so I would definitely say our high uninsured rate. I think that's the number one barrier.” [Clinic level]
		“Our volume of uninsured patients is another barrier.” [System level]
Restrictive health care policies	2	“I just found out that we couldn't get Title X because just the Health Department has it. I thought maybe we could heed in on Title X and that way, we'd be covered, but unfortunately not.” [Clinic level]
		“I think that healthcare environment in general and insurance coverage of contraception.” [System level]
Sustainability of Trainings		
Facilitators	Total (*N* = 34)	Representative quotations

Sustainability of trainings		
Facilitators	Total (*N* = 34)	Representative quotations
Continue to provide staff training	10	“We're still building in training for our providers, making sure that everybody gets the proper training on contraceptive counseling, as well as information on the [IUDs and implants]”. [Clinic level]
		“We will continue to educate our staff and our team with any means that we can.” [System level]
Training provided by experienced staff within system	4	“Our providers will already be trained and as we get more providers in, those providers that have already been here and have already been trained, can be preceptors for those new providers.” [System level]
		“We're going to be having a training room and we ordered four IUD simulators. [Health Care Center] will teach IUD and [trainer] will teach [the implant], so we can do our own training in house.” [Systems level]

Perceived value of training		
Facilitators	Total (*N* = 34)	Representative quotations
Expanded training opportunities is critical to increased access	13	“The trainings because without the trainings we couldn't be where we are today.” [Clinic level]
		“I would say education was probably the biggest for us.” [System level]
Applicability of training content	10	“[Trainings] empowered us to do our job even better. Every time we end up going, we get new information.” [Clinic level]
		“It's all very practical and easy to apply.” [System level]
Enhanced inter clinic networking	6	“…To interact also with other clinics and you bounce ideas off of them and you see what they're doing and what's working and what's not working.” [Clinic level]

FQHC, federally qualified health center.

Regarding funding, the availability of the 340B drug pricing program was noted as a facilitator of sustainability. The pricing program was noted “to provide contraceptives to clients whose insurance does not cover it.” Similarly, respondents emphasized continuing to seek other sources of external funding to provide same-visit IUD and implant provision and to order and stock devices on-site.

In addition, increasing the number of patients enrolled in Medicaid, enrolling patients in patient assistance programs, and offering a sliding scale fee for uninsured patients were all highlighted as sustaining factors to increased access. One individual at the system level noted: “You're going to get the reimbursement from the insurance companies and from Medicaid. We're trying to get everybody that will qualify on Medicaid, at least for family planning if they don't qualify for the full Medicaid.”

Plans to continue to provide staff training and to facilitate transfer of knowledge from trained staff within the FQHC system were also highlighted as sustaining factors. “For example, our providers will already be trained and as we get more providers in, those providers that have already been here and have already been trained, can be preceptors for those new providers.”

Respondents noted that expanded training opportunities are critical to increased access. One respondent emphasized, “…without the trainings we couldn't be where we are today.” Other perceptions of sustaining factors included: the applicability of training content to practice and enhanced networking among clinics.

Barriers to sustainability discussed were the high cost of devices, high rate of uninsured patients, and restrictive health care policies, such as FQHC clinics not being Title X recipients in SC, and limited insurance coverage for contraception.

## Discussion

In this study, we examined perceptions of FQHC clinic- and system-level staff participating in the SC Choose Well initiative regarding organizational factors that facilitated or posed challenges to access to IUDs and implants during the first 2 years of the initiative's implementation. Staff perceptions of the early impact of the Choose Well initiative on contraceptive access at their clinics were highly positive. Staff noted how the initiative had influenced multiple clinical and administrative policies/practices related to contraceptive access. This suggests that statewide contraceptive access initiatives that remove cost barriers to care, while building the capacity of safety-net clinics to provide contraceptive services, have the potential for advancing equitable access to care.

Challenges remain, particularly related to sustaining efforts beyond the initiative's funding period and ensuring patient-centered care. On-going evaluation of Choose Well will examine the impact of the initiative at the clinic, population, and policy levels.

Findings contribute novel and relevant nuance about access to IUDs and implants among non-Title X funded safety-net clinics in SC. Notably, components of access extend beyond clinical factors, such as on-site stocking of IUDs and implants and availability of trained and willing providers, into administrative factors, such as organizational buy-in, enhanced workflow processes, electronic infrastructure enhancement, and billing and coding support.

Respondents emphasized the benefits of external funding for IUDs and implants to reduce the cost barrier to patients, consistent with previous studies.^27^ In addition, themes suggest that an increase in funding for IUDs and implants contributed to clinics' ability to stock devices and provide same-visit placements, which contributed to expanded access, as noted previously.^4,28–31^ There are varying opinions among clinicians regarding the importance of same-visit IUD and implant placement,^32^ but respondents in our study emphasized that having the financial means to stock devices and provide same-visit placement procedures positively impacted access to IUDs and implants.

In addition, increased capacity for and enhanced contraceptive counseling was noted as a primary facilitator of access to IUDs and implants, as it increased patient education and expanded perspectives, such as gaining new viewpoints, examining personal prejudices, and increasing comfort level with discussing contraceptives, among reproductive health providers.

Overall, enhanced training opportunities were perceived to have increased capacity for contraceptive service provision, consistent with previous findings among FQHC clinics.^3^ Although training for IUD and implant provision is recommended for a breadth of providers, including midwives, advanced practice nurses, family medicine providers,^33^ and adolescent and pediatric providers,^34^ it is not always included in programs outside of Obstetrics and Gynecology specialties.^33^ One sustainability strategy emerging from our interviews is the continuous provision of staff training through implementing preceptorship programs with already trained providers.

In terms of administrative factors, buy-in and engagement among staff and leadership was the most noted facilitator to increased access to IUDs and implants. Enhanced workflow processes and infrastructure support and improvements in billing and coding were similarly noted to facilitate access to IUDs and implants in our study. Enhanced interclinic networking was also emphasized.

Respondents noted challenges to increasing access to contraception in their clinics, which related primarily to lack of buy-in to organizational change among staff. The systems changes required to implement medical innovations, such as integration of contraceptive care, require administrative support and buy-in from multiple levels within the organization.^35^ Optimizing the impact of statewide initiatives on clinical care among FQHCs necessitates attention to securing buy-in through on-going engagement with leadership, staff, and partnerships with other organizations to support internal influence.^35^ A few respondents also noted challenges related to provider factors (comfort, beliefs).

Providers averse to offering IUDs and implants due to their beliefs and perceptions may hinder patients' access to the full range of method options,^29^ suggesting the importance of on-going education and support for providers. A few respondents also mentioned workforce turnover and on-going issues with billing and coding. Incorrect billing and coding for medical services may lead to denied claims, which threaten the financial capacity for the clinic to maintain a stock of devices.^19^ Coverage and reimbursement policies across insurance plans complicate billing and coding, and on-going technical assistance for clinics in this area is needed.

Considering the lack of Title X funding for FQHC clinics in SC, sustaining factors must be considered. Structural factors related to cost of contraceptive services/devices and insurance coverage emerged as prevalent challenges to sustainability. 340B drug pricing was noted as one solution. Previous research indicates variation among FQHC clinics in utilizing 340B drug pricing, with clinics that have on-site pharmacies or receive Title X funding more likely to engage with 340B drug pricing,^19^ indicating opportunities for increasing clinic participation in this program.

Another suggested strategy for sustainability is facilitating patient enrollment in Medicaid. Nationwide, health centers serve about 16% of Medicaid beneficiaries, and Medicaid payments constitute nearly 40% of the revenue of FQHC clinics.^10^ Beyond these approaches, our findings reinforce the need for expanded insurance coverage, particularly in states such as SC that did not expand Medicaid under the Patient Protection and Affordable Care Act (ACA), as well as for supportive reimbursement policies to increase access to the full range of contraceptive methods.^20^ In addition, an important consideration for contraceptive access initiatives is ensuring patient-centered care, including best practices in contraceptive counseling that prioritize patients' needs and preferences and respect patients' reproductive autonomy.^36–41^

This study is not without limitation. While this study included a large number of respondents (*N* = 34), some members of the participant pool declined to participate, which might have contributed to potential response bias skewed toward participants eager to participate or with positive opinions about IUD and implant access. Due to the nature of qualitative research, researcher bias was a potential limitation of this study. To account for such bias, the study team was composed of a diverse group of people, and a team-based analysis approach was used. Data were double coded using an 80% or greater threshold for interrater reliability, and all differences were consensus coded.

Furthermore, as this study focused on FQHC clinics participating in the Choose Well initiative in SC, these findings may not be generalizable to other FQHCs, but rather may inform future efforts to expand contraceptive access at non-Title X clinics. The study was limited to assessing perceptions/opinions of clinic staff and did not measure clinic practices or other dimensions of access to care quantitatively or directly. On-going clinic surveys and analysis of population-level data will expand and further inform the evaluation.

## Conclusion

Strengthening the family planning safety-net clinic network remains a priority at the state and national levels. Given the volatility of federal funding sources,^42^ other funding mechanisms are crucial to bolster contraceptive availability among safety-net clinics that do not receive Title X funding. Statewide contraceptive access initiatives that combine funding for contraceptives with training and capacity building have the potential to support contraceptive policies and practices at FQHC clinics. By increasing access to a full range of contraceptive options and prioritizing patient-centered counseling, these initiatives could help ensure contraceptive choice and reproductive autonomy, improve health outcomes, and reduce inequities.

Based on the interview findings, the Choose Well initiative was perceived by clinic staff to have contributed to increased provision and access to contraception at participating FQHC clinics in SC through changes to clinic-level policies and practices and enhanced counseling. Staff also highlighted on-going challenges and perceived barriers to sustainability over time.

Optimizing the impact of Choose Well, and potentially of other contraceptive initiatives, requires coupling organizational change at the clinic/health system level with broader structural and policy changes to reinforce benefits of training and clinic enhancements. For example, state-level contraceptive access initiatives could advocate to strengthen health care delivery systems (through protecting contraceptive access under the ACA and restoring contraceptive access through expanded Medicaid programs) and support innovative policymaking and implementation of new delivery channels such as pharmacist prescribing.

## Abbreviations Used

FQHC: federally qualified health centers
IUD: intrauterine device
SC: South Carolina

## Appendix

**Appendix Table A1.** Sampling Approach and Sample Sizes for South Carolina FQHC Staff Interviews by Year and Level

FQHC systems	*N*	FQHC clinics	*N*
FQHC systems in SC that offered family planning services (2017)	20	FQHC clinics in SC that offered family planning services (2017)	123
FQHC systems participating in Choose Well in 2017	8	FQHC clinics participating in Choose Well in 2017	33
Of participating systems, FQHC systems invited to interview in 2018	8	Of participating clinics, FQHC clinics invited to interview in 2018	32
Of participating systems, FQHC system-level staff invited to interview in 2018 (*N* = 1–3 individuals per system)	11	Of participating clinics, FQHC clinic-level staff invited to interview in 2018 (*N* = 1–3 individuals per clinic)	38
Of those invited, FQHC systems interviewed in 2018	4	Of those invited, FQHC clinics interviewed in 2018	13
Of those invited, FQHC system-level staff interviewed in 2018	**6**	Of those invited, FQHC clinic-level staff interviewed in 2018	**13**
FQHC systems	*N*	FQHC clinics	*N*
FQHC systems in SC that offered family planning services (2018)	21	FQHC clinics in SC that offered family planning services (2018)	126
FQHC systems participating in Choose Well in 2018	16	FQHC clinics participating in Choose Well in 2018	57
Of participating systems, FQHC systems invited to interview in 2019	12	Of participating clinics, FQHC clinics invited to interview in 2019	39
Of participating systems, FQHC system-level staff invited to interview in 2019 (*N* = 1–3 individuals per system)	21	Of participating clinics, FQHC clinic-level staff invited to interview in 2019 (*N* = 1–3 individuals per clinic)	51
Of those invited, FQHC systems interviewed in 2019	7	Of those invited, FQHC clinics interviewed in 2019	14
Of those invited, FQHC system-level staff interviewed in 2019^a^	**8**	Of those invited, FQHC clinic-level staff interviewed in 2019^b^	**18**
Total system-level interviews in 2018 and 2019	**14**	Total clinic-level interviews in 2018 and 2019	**31**
Final system-level staff interviews in sample^a^	**11**	Final clinic-level staff interviews in sample^b^	**23**
Final no. of system-level and clinic-level staff interviews in sample 34			

The bold values show 1. the number of unique individuals that were interviewed in each year of the study; 2. the total number of interviews for both years; and 3. the total number of unique interviews across both years (as some people were interviewed in both years).

^a^ *N* = 3 were also interviewed in 2018 and counted once in final sample.

^b^ *N* = 8 were also interviewed in 2018 and counted once in final sample.

SC, South Carolina.

FQHC, federally qualified health center.

## References

[1] Frost JJ, Zolna MR, Frohwirth LF, et al. Publicly supported family planning services in the United States: Likely need, availability and impact, 2016. New York, New York: Guttmacher Institute, 2019:52. DOI:10.1363/2019.30830

[2] Potter JE, Stevenson AJ, Coleman-Minahan K, et al. Challenging unintended pregnancy as an indicator of reproductive autonomy. Contraception 2019;100:1–4. 30851238 10.1016/j.contraception.2019.02.005 PMC6919552

[3] Beeson T, Wood S, Bruen B, Goldberg DG, Mead H, Rosenbaum S. Accessibility of long-acting reversible contraceptives (LARCs) in Federally Qualified Health Centers (FQHCs). Contraception 2014;89:91–96. 24210278 10.1016/j.contraception.2013.09.014

[4] Bergin A, Tristan S, Terplan M, Gilliam ML, Whitaker AK. A missed opportunity for care: Two-visit IUD insertion protocols inhibit placement. Contraception 2012;86:694–697. 22770798 10.1016/j.contraception.2012.05.011

[5] Frost JJ, Gold RB, Bucek A. Specialized family planning clinics in the United States: Why women choose them and their role in Meeting Women's Health Care Needs. Womens Health Issues 2012;22:e519–e525. 23122212 10.1016/j.whi.2012.09.002

[6] Simmons M, Guerra-Reyes L, Meyerson B, Adams K, Sanders S. Exploring provider perspectives as barriers and facilitators to implementation of quality family planning recommendations at Title X Clinics: A qualitative study. Womens Health Issues 2016;26:628–633. 27745997 10.1016/j.whi.2016.08.005

[7] Hopkins B. Barriers to health care providers' provision of long-acting reversible contraception to adolescent and nulliparous young women. Nurs Womens Health 2017;21:122–128. 28388997 10.1016/j.nwh.2017.02.007

[8] Penchansky R, Thomas JW. The concept of access: Definition and relationship to consumer satisfaction. Med Care 1981;19:127–140. 7206846 10.1097/00005650-198102000-00001

[9] Beatty K, Smith M, Khoury A, Zheng S, Ventura L, Okwori G. Accessibility of Federally Funded Family Planning Services in South Carolina and Alabama. Prev Med Rep 2021;22:101343. 33767947 10.1016/j.pmedr.2021.101343 PMC7980054

[10] Wood S, Goldberg D, Beeson T, et al. Health centers and family planning: Results of a Nationwide Study. Washington, DC: The George Washington University, 2013:56. Available at: https://www.rchnfoundation.org/wp-content/uploads/2013/04/Health_Centers_and_Family_Planning-final-1.pdf Accessed November 11, 2021.

[11] Oglesby WH. Perceptions of and preferences for federally-funded family planning clinics. Reprod Health 2014;11(1):50. 24980897 10.1186/1742-4755-11-50 PMC4086278

[12] Wood S, Beeson T, Bruen B, et al. Scope of family planning services available in Federally Qualified Health Centers | Elsevier Enhanced Reader. Contraception 2014;89:85–90. 24176250 10.1016/j.contraception.2013.09.015

[13] Cottrell E, Darney BG, Marino M, et al. Study protocol: A mixed-methods study of women's healthcare in the safety net after Affordable Care Act implementation—EVERYWOMAN. Health Res Policy Syst 2019;17:1–10. 31186028 10.1186/s12961-019-0445-y PMC6558747

[14] National Family Planning and Reproductive Health Association. Washington, DC: The Title X family planning program in South Carolina. 2017. Available at: https://www.nationalfamilyplanning.org/file/impact-maps-2017/SC.pdf Accessed November 11, 2021.

[15] Mead KH, Beeson T, Wood SF, Goldberg DG, Shin P, Rosenbaum S. The role of Federally Qualified Health Centers in delivering family planning services to adolescents. J Adolesc Health 2015;57:87–93. 26095411 10.1016/j.jadohealth.2015.03.019

[16] Bornstein M, Carter M, Zapata L, Gavin L, Moskosky S. Access to long-acting reversible contraception among US publicly funded health centers. Contraception 2018;97:405–410. 29253581 10.1016/j.contraception.2017.12.010 PMC6750753

[17] de Bocanegra HT, Maguire F, Hulett D, Horsley K, Puffer M, Brindis CD. Enhancing service delivery through Title X funding: Findings from California. Perspect Sex Reprod Health 2012;44:262–269. 23231334 10.1363/4426212

[18] Luchowski AT, Anderson BL, Power ML, Raglan GB, Espey E, Schulkin J. Obstetrician–Gynecologists and contraception: Long-acting reversible contraception practices and education. Contraception 2014;89:578–583. 24656553 10.1016/j.contraception.2014.02.004

[19] Waxman Strategies. Factors influencing access to long-acting reversible contraceptives at Federally Qualified Health Centers. Washington, DC: Waxman Strategies, 2019.

[20] Ariyo O, Khoury AJ, Smith MG, et al. From training to implementation: Improving contraceptive practices in South Carolina. Contraception 2021;104:155–158. 33894253 10.1016/j.contraception.2021.04.016

[21] New Morning Foundation. Our Work—Choose Well SC. 2020. Available at: https://www.choosewellsc.org/our-work Accessed December 3, 2020.

[22] Gulzar L. Access to health care. J Nurs Scholarship 1999;31:13–19. 10.1111/j.1547-5069.1999.tb00414.x 10081206

[23] Goldberg DG, Wood SF, Johnson K, et al. The organization and delivery of family planning services in Community Health Centers. Womens Health Issues 2015;25:202–208. 25965153 10.1016/j.whi.2015.02.007

[24] Saldaña J. The coding manual for qualitative researchers. Sage Publications, Inc.; 2015:1–31. Available at: https://canvas.auckland.ac.nz/courses/1227/files/120502 Accessed October 19, 2020.

[25] Hruschka DJ, Schwartz D, St. John DC, Picone-Decaro E, Jenkins RA, Carey JW. Reliability in coding open-ended data: Lessons learned from HIV Behavioral Research. Field Methods 2004;16:307–331.

[26] Campbell JL, Quincy C, Osserman J, Pedersen OK. Coding in-depth semistructured interviews: Problems of unitization and Intercoder Reliability and Agreement. Sociol Meth Res 2013;42:294–320.

[27] Secura GM, Allsworth JE, Madden T, Mullersman J, Peipert JF. The Contraceptive CHOICE Project: Reducing barriers to long-acting reversible contraception. Am J Obstet Gynecol 2010;203:115.e1–e7. 20541171 10.1016/j.ajog.2010.04.017 PMC2910826

[28] Goodman S, Hendlish SK, Benedict C, Reeves MF, Pera-Floyd M, Foster-Rosales A. Increasing intrauterine contraception use by reducing barriers to post-abortal and interval insertion. Contraception 2008;78:136–142. 18672115 10.1016/j.contraception.2008.03.008

[29] Lotke PS. Increasing use of long-acting reversible contraception to decrease unplanned pregnancy. Obstet Gynecol Clin N Am 2015;42:557–567. 10.1016/j.ogc.2015.07.008 26598299

[30] Serpico JJ, Ricks JM, Smooth WG, Romanos C, Brook DL, Gallo MF. Access to single-visit IUD insertion at obstetrician-gynecology practices in Ohio: An audit study. Contraception 2020;102:190–194. 32450080 10.1016/j.contraception.2020.05.007

[31] Janiak E, Clark J, Bartz D, Langer A, Gottlieb B. Barriers and pathways to providing long-acting reversible contraceptives in Massachusetts Community Health Centers: A Qualitative Exploration. Perspect Sex Reprod Health 2018;50:111–118. 29940086 10.1363/psrh.12071

[32] Biggs MA, Arons ARA, Turner R, Brindis CD. Same-day LARC insertion attitudes and practices. Contraception 2013;88:629–635. 23809277 10.1016/j.contraception.2013.05.012

[33] Hathaway M, Torres L, Vollett-Krech J, Wohltjen H. Increasing LARC utilization: Any woman, any place, any time. Clin Obstet Gynecol 2014;57:718–730. 25314089 10.1097/GRF.0000000000000071

[34] Potter J, Koyama A, Coles MS. Addressing the challenges of clinician training for long-acting reversible contraception. JAMA Pediatr 2015;169:103–104. 25436764 10.1001/jamapediatrics.2014.2812

[35] Goodman S, Gordon R, Eckhardt C, Osborne S, Grossman D, Spiedel JJ. Beyond education and training: Making change stick. Contraception 2009;79:331–333. 19341842 10.1016/j.contraception.2009.01.018

[36] Dehlendorf C, Krajewski C, Borrero S. Contraceptive counseling: Best practices to ensure quality communication and enable effective contraceptive use. Clin Obstet Gynecol 2014;57:659–673. 25264697 10.1097/GRF.0000000000000059 PMC4216627

[37] Gomez AM, Fuentes L, Allina A. Women or LARC first? Reproductive autonomy and the promotion of long-acting reversible contraceptive methods. Perspect Sex Reprod Health 2014;46:171–175. 24861029 10.1363/46e1614 PMC4167937

[38] Holt K, Reed R, Crear-Perry J, Scott C, Wulf S, Dehlendorf C. Beyond same-day long-acting reversible contraceptive access: A person-centered framework for advancing high-quality, equitable contraceptive care. Am J Obstet Gynecol 2020;222(4 Supplement):S878.e1–S878.e6. 31809706 10.1016/j.ajog.2019.11.1279

[39] Horvath S, Bumpus M, Luchowski A. From uptake to access: A decade of learning from the ACOG LARC Program. Am J Obstet Gynecol 2019;222:S866–S868. 31794720 10.1016/j.ajog.2019.11.1269

[40] Fox E, Reyna A, Malcolm NM, et al. Client preferences for contraceptive counseling: A systematic review. Am J Prev Med 2018;55:691–702. 30342632 10.1016/j.amepre.2018.06.006 PMC6655529

[41] Zapata LB, Pazol K, Dehlendorf C, et al. Contraceptive counseling in clinical settings: An updated systematic review. Am J Prev Med 2018;55:677–690. 30342631 10.1016/j.amepre.2018.07.006 PMC6613590

[42] Zolna MR, Finn S, Frost J. Estimating the impact of changes in the Title X network on patient capacity. Guttmacher Institute. 2020. Available at: https://www.guttmacher.org/sites/default/files/article_files/estimating_the_impact_of_changes_in_the_title_x_network_on_patient_capacity_2.pdf Accessed October 20, 2020.

